# Transbronchial cryobiopsy in unexplained, severe ARDS: a single center retrospective case series

**DOI:** 10.1186/s12890-022-02296-1

**Published:** 2023-01-05

**Authors:** Stephan Eisenmann, Nina Lambrecht, Linda Dießel, Christin Busse, Sebastian Nuding, Alexander Vogt

**Affiliations:** 1 Department of Internal Medicine I, University Hospital Halle, Ernst-Grube-Strasse 40, 06120 Halle, Germany; 2 Department of Pathology, University Hospital Halle, Magdeburger Strasse 2, 06112 Halle, Germany; 3 Department of Internal Medicine, IIIUniversity Hospital Halle, Ernst-Grube-Strasse 40, 06120 Halle, Germany

**Keywords:** Transbronchial lung biopsy, Cryobiopsy, ARDS, ECMO

## Abstract

**Background:**

Acute respiratory distress syndrome (ARDS) deptics an acute form of lung infjury with often severe respiratory impairment that requires invasive mechanical ventilation. Since ARDS can be caused by several distinct etiologies, correct characterization is desired and frequently challenging. Surgical lung biopsy was previously reported to be of additive value. We describe our institutional experience using transbronchial cryobiopsy (TBCB) for further characterization of severe and unexplained ARDS cases.

**Case presentation:**

We retrospectively collected data of TBCB in patients with unexplained ARDS, whether with or without ECMO-support. Between 2019 and 2020 TBCB was performed in eight patients. Decision for the intervention was decided in multidisciplinary discussion. Five patients were treated with ECMO. The median duration of invasive ventilation before TBCB was 24 days. TBCB was performed in one segment, that was prophylactically occluded by Watanabe spigot or swab after the procedure. Histology results and their contribution to further therapeutic decisions were analyzed. Histology revealed five diffuses alveolar damage, one acute fibrinoid organizing pneumonia, one cryptogenic organizing pneumonia and one lung cancer. All results contributed to the decision of further management. While no pneumothorax or severe endobronchial bleeding occurred, two delayed hematothoraces needed surgical treatment. No patients died due to TBCB.

**Conclusion:**

TBCB is feasible in ARDS even during ECMO treatment. Histologic results can play a significant role in therapeutic and ethic discussion to guide the patients’ care. Side effects should be considered and monitored.

## Background

Acute respiratory distress syndrome (ARDS) depicts an acute form of lung injury with often severe respiratory impairment that requires invasive mechanical ventilation. The diagnosis consists of clinical and radiological features. Several aetiologies of ARDS can be distinguished, the respective underlying cause influences the individual treatment strategy. Correct identification and early treatment are crucial for ARDS outcome [1].

Beside identification of patients’ intrinsic risk factors a multi-modal diagnostic approach is essential. Based on radiological criteria methods of interventional bronchology might be of additional use, if necessary. Tissue based diagnosis was described as helpful when performed by open lung biopsy but with relevant amount of side effects [2]. Bronchoscopic tissue collection by forceps biopsy in interstitial lung disease (ILD) is reported to have only a small diagnostic yield [3]. Therefore, other methods need to be investigated.

We describe our experience with transbronchial cryobiopsy (TBCB), a method that was introduced into the diagnostic workup of unexplained interstitial lung disease (ILD), in our patients with unexplained severe ARDS with dependency on mechanical ventilation with or without extra corporal membrane oxygenation (ECMO).

## Case presentation

We retrospectively analyzed our single center case series of patients suffering from unexplained hypoxemic respiratory failure that meet the Berlin criteria of acute respiratory distress syndrome (ARDS) from June 2019 to July 2022. Our tertiary referral center provides a specialized extra-corporal membrane oxygenation (ECMO) program for an area of approximately three million inhabitants. The department of interventional pulmonology has profound knowledge in TBCB either in the work up of ILD or in diagnosis and therapy of lung cancer. As our thoracic surgery team works for several hospitals in the region and has limited ressources for elective diagnostic procedures we usually aim to achieve diagnosis by bronchoscopy first, whenever possible.

Inclusion criteria were (1) underlying unexplained ARDS based on Berlin definition with clinical and radiological interpretation, (2) current high resolution computed tomography (HR-CT) of the lung, (3) exclusion of a persistent or predominant infectious ARDS cause based on previous investigation including bronchioalveolar lavage (BAL), (4) multidisciplinary discussion (including radiologist, pathologist, intensive care physician, interventional pulmonologist and thoracic surgeon) requested additional tissue sampling to understand the cause and prognosis of the individual patients’ ARDS, (5) individual informed consent to the TBCB given by legal representatives and (6) no severe additional condition that does not allow the performance of TBCB. Treatment with extracorporal membrane oxygenation (ECMO) was not an exclusion criterion. Effective anticoagulation therapy was paused or reduced, if possible.

**Fig. 1:**
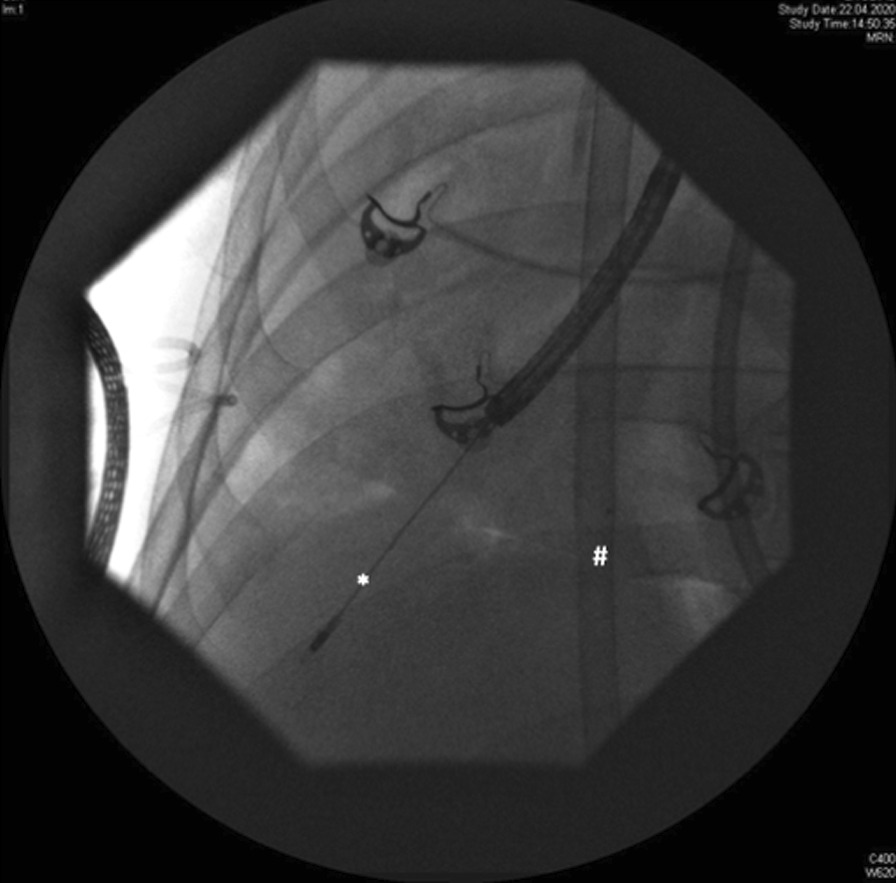
Transbronchial cryobiopsy (1.9 mm flexible cryoprobe, ERBE, Tuebingen, Germany, *) in the right lower lobe. ECMO cannula located in the inferior cava vein (#)

TBCB was performed as previously recommended [4, 5]. Shortly, a flexible bronchoscope (Olympus, 2.8 mm working channel, Japan) was inserted during rigid bronchoscopy (Storz, Tuebingen, Germany) or the established endotracheal tube. Ventilatory support was dependent on the respective approach. A flexible 1.9 mm cryoprobe (ERBE, Tuebingen, Germany) was inserted through the working channel into the subpleural zone of the lung periphery, controlled with fluoroscopy whenever possible (Fig. 1). Biopsies were taken only from one pulmonary segment to minimize bleeding complications. The biopsy location was previously selected with regard to the HR-CT and simple endoscopic access. The maximum freezing time was agreed with five seconds, a total amount of 3 visible tissue specimen were extracted and placed into formalin. Tissue specimen were processed and investigated in the Institute of Pathology of the university of Halle-Wittenberg.

After every biopsy segmental suction with the wedge-positioned bronchoscope was done for at least one minute. A prophylactic balloon blockade during bronchoscopy was not regularly used. To avoid deleyed endobronchial bleeding especially in case of therapeutic anticoagulation after TBCB the selected segment was blocked with swabs or Watanabe-Spigot of 6 or 7 mm diameter (Novatech, Lyon, France), depending on the individual anatomy for 48 h, that were removed in a consecutive flexible bronchoscopy without complications.

Pneumothorax exclusion was performed by transthoracic ultrasound (TUS) immediately after the procedure and at least twice during the consecutive 6 h to monitor delayed pneumothorax and pleural effusion.

Indication for the tissue collection, the assessment of benefit/risk balances and consecutive analysis of the individual results were discussed at the in-house multi-disciplinary panel, which consists of delegates of the departments of pathology, interventional pulmonology, intensive care medicine, radiology and thoracic surgery. Open lung biopsy was intended in case of non-diagnostic TBCB.

Patients’ data were de-identified, the informed consents for the procedures were given by dedicated representatives after individual risk-based information. The retrospective analysis was approved by the ethics committee (IRB-No. 2020–155) of the Martin Luther University Halle-Wittenberg.

From July 2019 to July 2022 eight patients (five males, three females) with a median age of 68 (37;83) years suffering from unexplained ARDS underwent a transbronchial lung-cryobiopsy. The median number of days of ventilator dependency was 23 (17, 48). Patients’ individual data are summarized anonymously in Table 1. One patient had known mild lung emphysema, whilst the rest had no prior pulmonary disease. The assumed ARDS-cause was idiopathic or unknown (n = 4), infection with SARS-CoV-2 (n = 2), RSV pneumonia (n = 1) and bacterial pneumonia (n = 1). With intention to treat post-inflammatory ARDS all patients were effortlessly treated with systemic steroids before indication of the cryobiopsy was discussed.

**Table 1:** Patient and procedure details. Numbers are median or absolute count

Median Age (years)	68 (37;83)
Male/Female	5/3
ECMO therapy	5
Median days on mechanical ventilation/ECMO before TBCB	23/10
Median SOFA Score	13 (10;20)
Median APACHE II Score	33 (22;41)
Sufficient histopathology (n)	8/8
Impact on clinical management (n)	8/8
Biopsy location	LB5 n = 3 Left lower lobe n = 1 RB3 n = 1 RB8 n = 2 RB9 n = 1
Severe adverse event	Endobronchial bleeding n = 0 Hematothorax due to pleural defect in VATS n = 1 Hematothorax without pleural defect n = 1 Pneumothorax n = 0 Respiratory deterioration n = 0

*ECMO* extracorporal membrane oxygenation, *TBCB* transbronchial cryobiopsy, *VATS* video assisted thoracoscopy

There was no clinical and laboratory evidence for active infection at the time of TBCB. Bronchioalveolar lavage (BAL) was done in every patient at least two days prior to the biopsy and did not reveal relevant bacterial load or immunological explanation. Extended laboratory testing excluded an underlying autoimmune disorder. Transbronchial forceps biopsy was earlier done in one patient without representative alveolar tissue.

Five patients were dependent on veno-venous ECMO support at the time of TBCB. The PTT-controlled anticoagulation was reduced in the periinterventional hours to a target of 50 s. Three patients did not depend on ECMO at the moment of bronchoscopy. None of the patients had an additional indication for therapeutic heparinization.

Biopsy locations were distributed equally between the right and the left lung. Five patients were biopsied with fluoroscopy, in three patients the transport to the endoscopy unit was impossible because patient transfer posed a relevant individual risk. All patients received three biopsies, and no intervention was impaired by immediate endobronchial bleeding after the initial suction in wedged position.

**Fig. 2:**
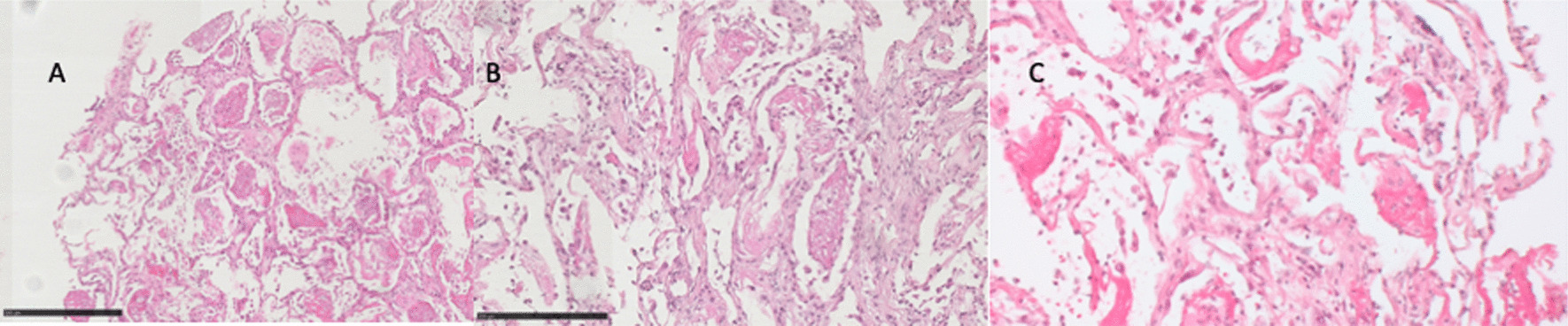
Diffuse alveolar damage—Hamman Rich Syndrome (A: 80x; B: 170x; C: 200x). **a** Initial formation of airspace granulation tissue plugs in alveolar ducts. **b** Accumulation of intra-alveolar macrophages. **c** Organizing /proliferative phase AIP/DAD with hyaline membranes and the very large

**Fig. 3:**
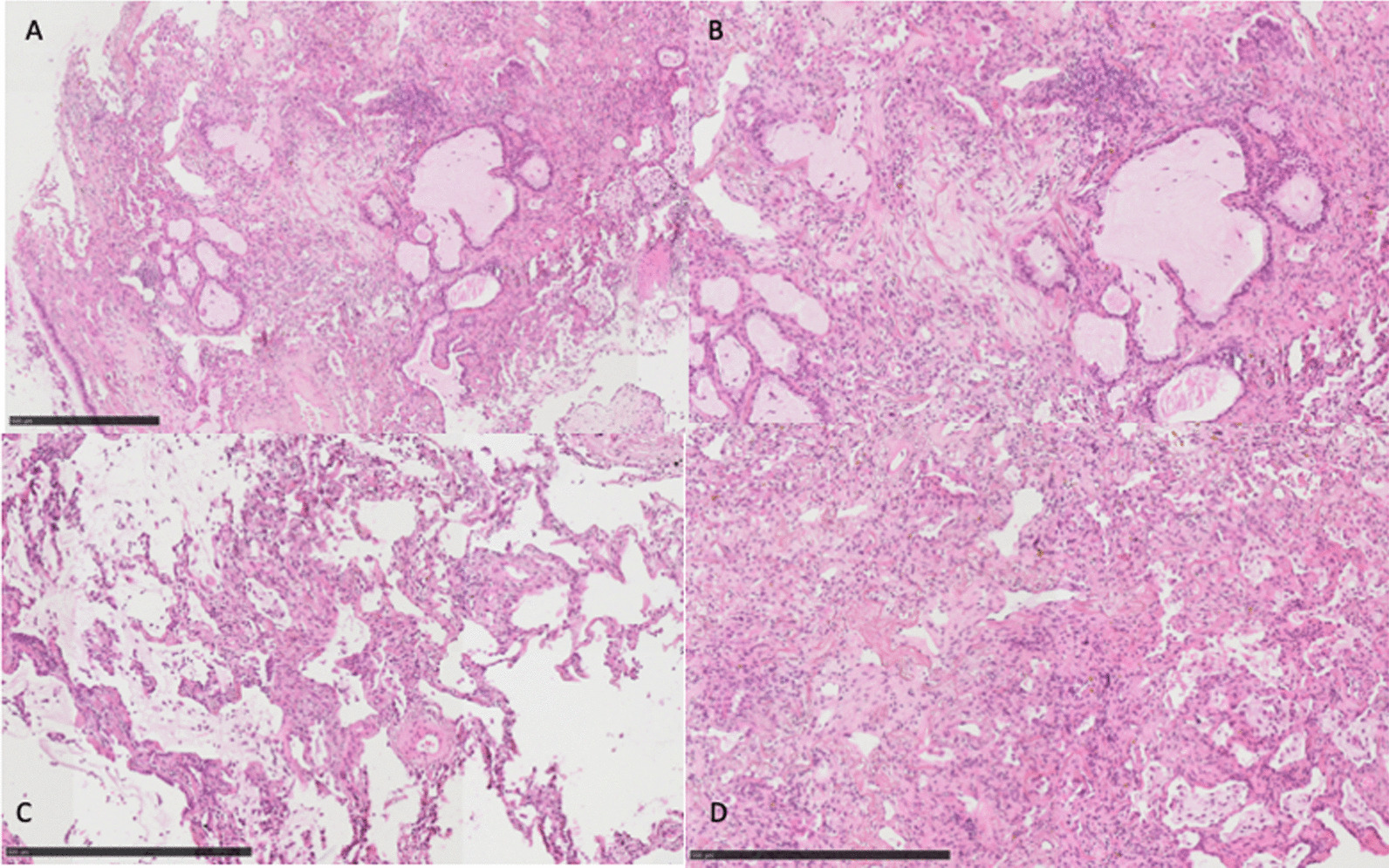
COVID-ARDS (A: 70x, B: 100 x, C&D: 120x). **a**, **b** Fibrotic lung parenchyma with mucous metaplasia, chronic interstitial inflammation and centered myofibroblastic proliferations. **c** Fibrotic parenchyma with intraalveolar edema. **d** Fibrotic parenchyma with chronic interstitial inflammation and accumulated macrophages

Histopathological evaluation revealed representative, diagnostic material in all eight patients: Five diffuse alveolar damage (DAD, Fig. 2), one acute fibrinoid organizing pneumonia (AFOP), one cryptogenic pneumonia (COP, Fig. 3) and one bronchoalveolar carcinoma (NSCLC), respectively. In three patients an individual therapeutic intervention based on the histological findings of AFOP, COP and NSCLC was initiated. In five cases the histology results with DAD offered no additional treatment opportunity but were important to guide the upcoming decisions regarding limitation of intensive care.

No pneumothorax occurred within the postinterventional period. In patient #1 increasing pleural fluid was detected with TUS, a pleural tube placement showed a hematothorax that needed a single surgical intervention (video assisted thoracoscopy). Here a pleural defect was identified as bleeding source. Red blood cell transfusion was necessary. Patient #4 experienced a delayed hematothorax with necessary thoracotomy 48 h after TBCB. Diffuse pleural bleeding was described and treated with pleural packing, transfusion and correction of coagulation factors. However, these two severe complications occurred only in patients who were biopsied without fluoroscopy. Both of these patients were on ECMO treatment.

In the other six cases no severe complications occurred. Prophylactic blocking devices were easily placed and removed consecutively. No severe endobronchial bleeding that might have caused additional maneuvers or earlier termination happened immediately during the TBCB.

None of the patients survived their intensive care admission due to ongoing medical conditions, but no patient deceased with direct correlation to the TBCB. The patient with SARS-CoV2-ARDS received a post-mortem examination that confirmed the histological diagnosis of COP gained by TBCB. The patient with new diagnosed lung cancer received a combined immune-chemotherapy, which lead to initial improvement of oxygenation. With a delay of ten days that patient died due to nosocomial infection during cell depletion phase. The remaining seven patients received a limitation of ICU treatment after interdisciplinary ethic council.

## Discussion and Conclusion

Transbronchial cryobiopsy (TBCB) is feasible and of additional diagnostic benefit in patients that suffer from severe ARDS even during ECMO treatment. In our case series TBCB allowed further characterization of their unexplained ARDS and lead to change of the clinical management in all eight patients.

TBCB has been extensively analyzed especially in the diagnostic workflow of ILD. In this context it is broadly accepted that TBCB has moderate correlation when compared to surgical lung biopsy, but TBCB does increase confidence of MDD diagnosis when surgical lung biopsy is not available [6, 7]. Compared to surgical lung biopsy the lower rate of complications of TBCB and the comparable diagnostic yield is advantageous.

Recently TBCB has been reported in two retrospective series in a small number of patients with acute hypoxemic respiratory failure (AHRF) and ARDS without severe adverse events. However, these patients were investigated at a quite early moment after hospital admission, there is no information about the severity of the individual respiratory situation, especially need for mechanical ventilation [8–10].

We usually do not reach for tissue collection in an early ARDS, especially when no previous pulmonary pathology is known and usually an infectious cause might be assumed. In ARDS or AHRF tissue acquisition with open lung biopsy alone or in combination with BAL and lung forceps biopsy was reported to have a relevant impact to the diagnostic work up and change the treatment in up to 73% of the patients. It is however connected to a relevant number of severe adverse events and procedure related deaths [2]. In this particular patient cohort this is difficult to compare. Time to rapidly find an exact diagnosis is limited and usually does not allow several attempts of interventional methods. When tissue collection seems necessary the balance between a method with high diagnostic yield and expectable complication rate at the lowest possible level is essential. TBCB should be considered, when surgical lung biopsy is not consented or not warranted for any reason and skills with TBCB are already established.

Because of the need for diagnostic precision on one hand and manageable complications on the other hand, we decided not to perform TBFB regularly prior to TBCB. The TBFB prior to TBCB performed in one single patient did not reveal any relevant diagnostic information.

There is still debate on the procedural settings in TBCB [5]. Further studies have to contribute to this discussion. Transbronchial biopsy, even TBCB, has never been reported under ECMO. Also, open lung biopsy during ECMO treatment has never been reported before. As this seems to be a situation with high clinical need, we have shown that TBCB might be feasible with an acceptable risk profile.

We acknowledge the relevant risk profile in our patients, but the side effects could be handled consecutively. However, complications need to be effectively monitored and treated, if necessary. Prophylaxis of endobronchial haemorrhage with inserted blocking devices should be done especially when effective anticoagulation cannot be withdrawn. We did not work with a prophylactic balloon blockade, which is recommended during or immediately after TBCB especially when experience with the procedure is lacking or general endotracheal intubation is not performed [5]. We took into account an increased risk of delayed bleeding after restart of anticoagulation therapy especially in the patients on ECMO and therefore decided to regularly place Watanabe Spigots with a size of 6 mm or swabs instead of blocking ballons. Spigots and Swabs do not cause airway leakage like balloons and are easy to remove later on. As we experienced two severe complications in patients on ECMO where fluoroscopy was logistically impossible we postulate that this item should be mandatory to increase the periprocedural safety. When fluoroscopy is impossible to provide, TBCB especially in ARDS patients should not be performed.

Surveillance and treatment of procedure related side effects are essential. TUS has been reported to be an excellent tool for either exclusion of pneumothorax directly or delayed after a broad range of bronchoscopic interventions [11] or but also with exclusive focus on TBCB [12, 13], and is recommended to be used as first method for pneumothorax exclusion after procedures with increased risk for pneumothorax in the intensive care unit setting when compared to delayed chest X-ray [14]. In the one patient with pleural defect, the absence of pneumothorax might be explained due to stiffness of the lung tissue but is not generalizable.

We scheduled the biopsies quite late in the patients’ clinical course. The earlier cited studies reported performance of TBCB earlier in diagnostic workup / clinical course. This was however impossible in our patients since they were treated at later time of disease in our center. Whether an earlier transbronchial biopsy would have changed the clinical course remains speculative. This should be considered if even the initial presentation cannot be explained by infection or other common causes of ARDS. We used TBCB to evaluate the prognosis of the pulmonary damage. The suggested and reasonable diagnostic workflow describes tissue collection when previous steps including BAL could not elucidate the cause of the unexplained ARDS [1]. Earlier tissue characterization of unexplained ARDS and subsequent prognostication will help in difficult therapeutic decisions and better allocate limited ICU resources. With respect to the potential severe complications TBCB should only be performed when the patients’ individual constitution would also allow open lung biopsy with VATS.

Limitations of this report are due to the small number of patients and the missing control group. Results are not generalizable, prospective clinical studies should be scheduled to increase knowledge about the value of TBCB in this group of patients with enormous clinical need for the timely diagnosis.

## Conclusion

Transbronchial cryobiopsy (TBCB) is feasible and of additional diagnostic value in patients that suffer from unexplained ARDS, even when under ECMO support. When following the general recommendations for TBCB and ensuring close post-interventional monitoring, side effects and complications might be acceptable and manageable.

## Abbreviations

AFOP: Acute fibrinoid organizing pneumonia
AHRF: Acute hypoxemic respiratory failure
ARDS: Acute Respiratory Distress Syndrome
BAL: Bronchoalveolar Lavage
COVID: Corona Virus Disease
COP: Cryptogenic organizing pneumonia
DAD: Diffuse alveolar damage
ECMO: Extracorporal membrane oxygenation
HR-CT: High resolution computed tomography
ILD: Interstitial Lung Disease
SARS: Severe adult respiratory syndrome
TBCB: Transbronchial cryobiopsy
TBFB: Transbronchial forceps biopsy
